# Quality of life and metabolic status in mildly depressed patients with type 2 diabetes treated with paroxetine: A double-blind randomised placebo controlled 6-month trial

**DOI:** 10.1186/1471-2296-8-34

**Published:** 2007-06-15

**Authors:** Maria Paile-Hyvärinen, Kristian Wahlbeck, Johan G Eriksson

**Affiliations:** 1 National Public Health Institute, Mannerheimintie 166, FIN-00300 Helsinki, Finland; 2 Psychiatric unit, Vaasa Central Hospital, FIN-65130 Vaasa, Finland; 3 National Research and Development Centre for Welfare and Health (STAKES) – P.O. Box 220, FI-00531 Helsinki, Finland; 4 Department of Public Health, University of Helsinki, PB 41, 00014 Helsinki, Finland

## Abstract

**Background:**

Depression is prevalent in people with type 2 diabetes and affects both glycaemic control and overall quality of life. The aim of this investigator-initiated trial was to evaluate the effect of the antidepressant paroxetine on quality of life, metabolic control, and mental well-being in mildly depressed diabetics aged 50–70 years.

**Methods:**

We randomised 49 mildly depressed primary care outpatients with non-optimally controlled diabetes to a 6-month double-blind treatment with either paroxetine 20 mg per day or matching placebo. Primary efficacy measurements were quality of life and glycaemic control. The primary global outcome of the study was defined as a 10 points improvement in the SF-36 quality of life score. The primary metabolic outcome of the study was defined as a 0.8%-units decrease in glycosylated haemoglobin A_1c_(GHbA_1c_). Psychiatric symptoms were assessed with the Hospital Anxiety and Depression Scale.

**Results:**

Six patients withdrew their consent before starting medication and six dropped out later in the study. We performed analysis of covariance with the baseline value as a covariate. Quality of life and glycaemic control as well as symptoms of depression and anxiety improved in both groups over the 6-month study period. After three months of treatment we found a statistically significant difference between the two treatment groups in GHbA_1c _(mean difference = 0.59%-units, p = 0.018) and in SF-36 score (mean difference = 11.0 points, p = 0.039). However, at the end of the study, no statistically significant differences between the treatment groups were observed. No severe adverse events occurred.

**Conclusion:**

This pragmatic study of primary care patients did not confirm earlier preliminary findings indicating a beneficial effect of paroxetine on glycaemic control. The study indicates that in pragmatic circumstances any possible benefit from administration of paroxetine in diabetic patients with sub-threshold depression is likely to be modest and of short duration. Routine antidepressant prescription for patients with diabetes and sub-threshold depressive symptoms is not indicated.

**Trial registration:**

Current controlled trials ISRCTN55819922

## Background

Type 2 diabetes, characterised by both insulin resistance and impaired insulin secretion, is a common disease with rapidly increasing prevalence worldwide [1,2]. Insulin resistance is one of the primary metabolic defects, both in the metabolic syndrome and in manifest type 2 diabetes. Therefore a main treatment target is to improve insulin sensitivity. The cornerstones of treatment are non-pharmacological i.e. exercise and improved dietary habits. Both weight loss and physical activity are known to improve insulin sensitivity [3]. Unfortunately, success of non-pharmacologic treatment is rare. Therefore other means to improve glycaemic control are urgently needed.

Depression is common among diabetics [4] and it has indeed been suggested that one possibility for pharmacological treatment of insulin resistance is the use of antidepressive agents such as the selective serotonin reuptake inhibitors (SSRIs). It has previously been shown that the SSRI fluoxetine lowers blood glucose levels in type 2 diabetics [5,6]. Fluoxetine has also been shown to promote weight loss [7], which in itself would improve insulin sensitivity. Interestingly, this effect of fluoxetine on insulin sensitivity also occurs independently of weight loss [8]. The mechanism behind this effect is not known.

The metabolic syndrome is characterised not only by insulin resistance but also by obesity, elevated blood pressure, dyslipidemia, and an increased risk for cardiovascular disease. Symptoms of depression among type 2 diabetics have been shown to correlate with glycaemic control although it is not clear whether this is due primarily to non-compliance with the anti-diabetic medication or to depression [9]. Therefore, in order to reduce the risk for cardiovascular diseases associated with the metabolic syndrome and type 2 diabetes, it is important to treat not only the metabolic derangements in type 2 diabetes but all risk factors accompanying the disease. It is of interest to see whether antidepressant drug therapy could prove beneficial for diabetics with regard to metabolic control as well as mental health.

The use of antidepressant medication has increased dramatically in many countries since the introduction of SSRIs [10]. A majority of prescriptions is done by general practitioners in primary care and many primary care patients have sub-threshold or mild depression [11].

We performed a small-scale single-blind paroxetine pilot study [12], and found that paroxetine had a beneficial effect on measures of insulin sensitivity. This motivated a further, longer and methodologically less biased study. The aim of our current study was to assess whether the SSRI paroxetine has beneficial effects on quality of life and overall metabolic control in a generalisable primary care sample of type 2 diabetics in the age range 50–70 years.

## Methods

Mildly depressed type 2 diabetes out-patients were invited to participate in this investigator-initiated 6-month trial. All subjects received standard diabetes treatment by their primary care physicians prior to and during the trial. Men and women between 50 and 70 years of age, who had been diagnosed with type 2 diabetes at least a year prior to study entry, were eligible for the study. They had to be on stable hypoglycaemic medication for at least three months before entering the study.

The inclusion criteria included non-optimal glycaemic control – defined as glycosylated haemoglobin A_1c _(GHbA_1c_) > 7.0 % – and mild depression, i.e. not more than six depressive symptoms according to Diagnostic and Statistical Manual of Mental Disorders, 4^th ^edition, (DSM-IV) criteria [13]. The participants were interviewed by one of the authors (M.P) who was trained to perform a psychiatric interview. Their mood and anxiety levels were evaluated using the clinician-rated Hospital Anxiety and Depression Scale (HADS) [14] and quality of life was assessed with the SF-36 questionnaire [15].

Subjects with a moderate to severe depression based on DSM-IV criteria were excluded as we considered it unethical to include more severely depressed patients because of the possibility of receiving placebo. Subjects were excluded if they had glaucoma [16] and if they were using warfarin [17] because of possible adverse effects of paroxetine in these conditions. Furthermore, subjects with major complications due to diabetes (e.g. major cardiovascular, renal or vascular disease, and blindness) and subjects who used any kind of antidepressants were also excluded.

Our primary outcomes were improvement of GHbA_1c _and the SF-36 quality of life score. A mean difference between the treatment groups of 0.8 %-units in GHbA_1c _was considered to be clinically significant and power calculation, assuming an alpha error of 5 % and a beta error of 20 %, indicated that 19 patients per group were required to evidence this difference. Allowing for a drop-out rate of 20 %, 50 patients were to be included in the study.

The randomisation was computerised and concealed to participants, investigators and treating physicians. The identical tablets were packed in identical vials by the hospital pharmacy according to the randomisation schedule. The allocation remained concealed throughout the trial. The investigators did not take part in the clinical treatment of the study participants.

At baseline and after 3 and 6 months the quality of life and mental status of the participants were evaluated using SF-36 and HADS. Body mass index (kg/m^2^) was calculated using body weight measured to the nearest kg and height to the nearest cm. At the same time points blood samples were drawn for the following analyses: serum glucose, GHbA_1c_, serum C-peptide and serum sex hormone binding globulin (SHBG). All measurements were made after an overnight fast.

At baseline, at 1, 3 and 6 months adverse events were registered and the following safety blood tests were taken: blood count, serum sodium, serum potassium and liver enzymes. Subjects were to be removed from the study if any safety tests showed abnormal results. We ensured compliance with the medication by counting the left over pills at each visit.

The study was approved by the Ethics Committee of Helsinki University Central Hospital. All participants signed an informed consent form and were not reimbursed for participating in the study.

Differences between the two treatment groups were assessed with analysis of covariance (ANCOVA), where baseline measurements were used as covariates. Changes from baseline were calculated with a paired samples t-test. Kolmogorov-Smirnov-test was used to test for normality of the data. All analyses were made with the SPSS 13.0 software.

## Results

**Figure 1:**
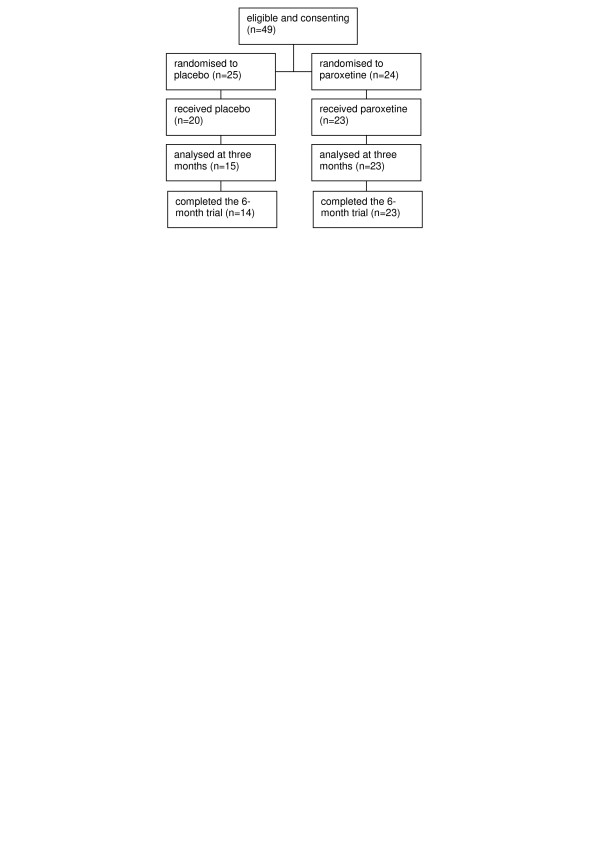
Flow chart of trial participants.

Seventy-two diabetic patients were interviewed in person and 23 of these did not meet the inclusion criteria for the study. The most common reason for exclusion was good glycaemic control indicated by a GHbA1c-value of 7.0 % or lower (n = 15). The remaining 49 subjects were randomly assigned to take either placebo (n = 25) or 20 mg paroxetine (n = 24) once a day in a double-blind fashion. Six subjects withdrew their consent before starting medication. Six participants dropped out later in the study, notably all of them allocated to the placebo group (Figure 1). Two subjects dropped out due to lack of positive sensations from the treatment. Other reasons for aborting were initiation of insulin treatment (n = 2), erectile dysfunction (n = 1) and hospitalisation due to cardiac event (n = 1).

Baseline characteristics of those 33 men and 10 women who entered the trial and received medication are given in Table 1. The two groups had similar baseline features and were representative of identified primary care diabetes patients. The subjects were in general obese with a non-optimal glycaemic control having mean fasting serum glucose > 8 mmol/l. All patients were on antidiabetic medication. Seven of the 43 participants received insulin treatment only and 17 used a combination of insulin and oral hypoglycaemic agents.

**Table 1:** Baseline characteristics of mildly depressed patients with type 2 diabetes who entered treatment.

	**Placebo (n = 20)**		**Paroxetine (n = 23)**	
	
	Mean	SD	Mean	SD
Sex (M/F)	16/4		17/6	
Age (years)	59.5	6.0	59.2	5.4
Body mass index (kg/m2)	32.0	5.3	31.7	5.5
**Metabolic parameters**				
GHbA1c (%)	8.7	1.3	8.5	0.9
Fasting serum glucose (mmol/l)	10.4	3.4	10.4	3.7
Serum C-Peptide (nmol/l)	0.69	0.53	0.74	0.91
Sex hormone binding globulin (nmol/l)	43.2	23.1	37.0	18.7
**Mental status**				
Hospital Anxiety and Depression Scale total score	15.7	5.5	14.0	5.2
Hospital Anxiety and Depression Scale depression score	8.4	3.4	7.3	3.4
Hospital Anxiety and Depression Scale anxiety score	7.3	3.0	6.7	2.6
SF-36 composite score	48.5	15.7	56.2	17.4

Baseline HADS scores indicated presence of mild depressive and anxiety symptoms. Ten (5 in the paroxetine group and 5 in the placebo group) of the 43 participants who started medication fulfilled DSM-IV criteria for a mild major depressive episode and the remaining 33 (18 in the paroxetine group and 15 in the placebo group) had sub-threshold depressive symptoms.

**Figure 2:**
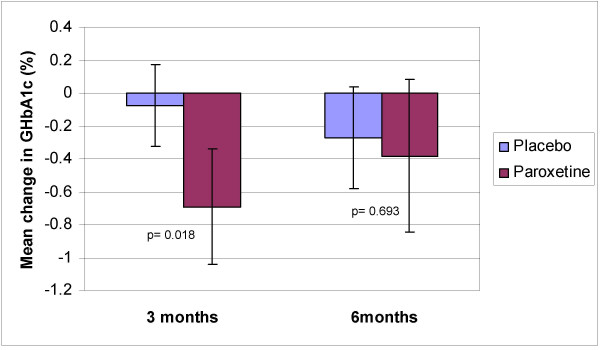
Mean changes in glycosylated haemoglobin A_1c _(GHbA_1c_) from baseline for type 2 diabetic subjects receiving placebo or paroxetine. Error bars represent 95% CI:s of means. P-values for difference between groups are calculated using ANCOVA with the baseline value as a covariate.

After three months there was a statistically significant improvement in GHbA_1c _in the paroxetine group (mean change from baseline = 0.69%-units, p = 0.001) but not in the placebo group (mean change = 0.11%-units, p = 0.33) (Figure 2). The difference in mean GHbA_1c _between groups was significant at three months. Controlling for baseline GHbA_1c_, HADS, SF-36 or BMI did not influence the result. At the end of the trial the change from baseline was no longer significant in the paroxetine group (mean change = 0.38%-units, p = 0.10) and there was no significant difference between groups (Table 2).

**Table 2:** Means (and 95% CI:s) of metabolic parameters for subjects who completed the study. Mean differences between groups were obtained with analyses of covariance with the baseline value as a covariate. Means at three and six months are estimated marginal means. GHbA1c = glycosylated haemoglobin A_1c_.

	**Placebo (n = 14)**	**Paroxetine (n = 23)**	**Placebo – Paroxetine**	
	Mean (95% CI)	Mean (95% CI)	Mean difference (95% CI)	p

**GHbA1c (%)**				
Baseline	9.0 (8.2 to 9.8)	8.5 (8.0 to 8.9)	0.54 (-0.24 to 1.31)	0.169
3 months	8.5 (8.2 to 8.9)	7.9 (7.6 to 8.2)	0.59 (0.11 to 1.07)	**0.018**
6 months	8.4 (7.9 to 8.9)	8.3 (7.9 to 8.7)	0.13 (-0.52 to 0.78)	0.693
**Serum glucose (mmol/l)**				
Baseline	10.7 (8.6 to 12.8)	10.4 (8.8 to 12.0)	0.27 (-2.27 to 2.81)	0.829
3 months	10.7 (9.8 to 12.5)	9.7 (8.2 to 11.1)	1.05 (-1.26 to 3.35)	0.362
6 months	10.8 (9.1 to 12.5)	10.2 (8.8 to 11.5)	0.59 (-1.56 to 2.74)	0.580
**Serum C-peptide (nmol/l)**				
Baseline	0.6 (0.3 to 0.8)	0.7 (0.5 to 0.9)	-0.15 (-0.46 to 0.15)	0.310
3 months	0.8 (0.6 to 0.9)	0.8 (0.7 to 1.0)	-0.04 (-0.25 to 0.16)	0.656
6 months	0.7 (0.6 to 0.9)	0.8 (0.7 to 0.9)	-0.04 (-0.21 to 0.14)	0.682
**Body mass index (kg/m2)**				
Baseline	31.1 (29.3 to 34.1)	31.7 (29.8 to 33.1)	-0.66 (-4.14 to 2.82)	0.704
3 months	31.1 (30.7 to 31.6)	31.0 (30.6 to 31.3)	0.15 (-0.44 to 0.73)	0.614
6 months	31.4 (30.9 to 31.9)	31.2 (30.8 to 31.6)	0.15 (-0.47 to 0.77)	0.629

**Figure 3:**
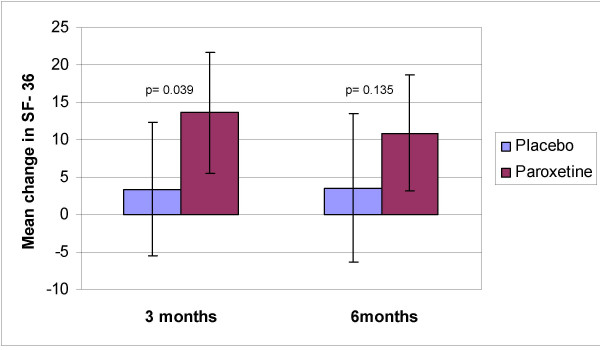
Mean changes in SF-36 quality of life score from baseline for type 2 diabetic subjects receiving placebo or paroxetine. Error bars represent 95% CI:s of means. P-values for difference between groups are calculated using ANCOVA with the baseline value as a covariate.

A significant improvement in overall quality of life, as measured by SF-36, was observed in the paroxetine group (Figure 3). After three months of treatment there was a statistically significant difference between the two treatment groups (mean difference = 11.0 points, p = 0.039) (Table 3). This difference remained significant after controlling for baseline SF-36 score, GHbA_1c _and BMI. At the end of the six month trial there was no statistically significant difference between the two treatment groups in SF-36 score (Table 3). However, the improvement from baseline was still statistically significant in the paroxetine group (Figure 3)

**Table 3:** Means (and 95% CI:s) of quality of life and mental health rating scores for subjects who completed the study.

	**Placebo (n = 14)**	**Paroxetine (n = 23)**	**Placebo-Paroxetine**	
	Mean (95% CI)	Mean (95% CI)	Mean difference (95% CI)	p

**SF-36**				
Baseline	51.2 (42.6 to 59.8)	56.2 (48.6 to 63.7)	-4.7 (-16.3 to 6.4)	0.381
3 months	59.1 (51.2 to 67.0)	70.1 (63.4 to 76.7)	-11.0 (-21.4 to -0.6)	**0.039**
6 months	56.9 (47.5 to 66.2)	65.8 (58.5 to 73.6)	-8.9 (-20.8 to 2.9)	0.135
**HADS total score**				
Baseline	15.8 (12.5 to 19.1)	14.0 (11.7 to 16.2)	1.8 (-1.9 to 5.6)	0.327
3 months	11.2 (9.0 to 13.5)	8.5 (6.6 to 10.4)	2.8 (-0.2 to 5.7)	0.066
6 months	12.1 (8.9 to 15.4)	10.2 (7.6 to 12.7)	1.9 (-2.2 to 6.1)	0.351
**HADS depression score**				
Baseline	8.2 (6.2 to 10.2)	7.3 (5.8 to 8.7)	0.9 (-1.4 to 3.4)	0.416
3 months	5.1 (3.8 to 6.3)	3.8 (2.7 to 4.9)	1.3 (-0.4 to 2.9)	0.129
6 months	6.2 (4.6 to 7.7)	5.5 (4.3 to 6.6)	0.7 (-1.2 to 2.7)	0.448
**HADS anxiety score**				
Baseline	7.6 (5.8 to 9.4)	6.7 (5.6 to 7.8)	0.9 (-1.1 to 2.8)	0.366
3 months	6.2 (4.8 to 7.6)	4.6 (3.5 to 5.8)	1.6 (-0.2 to 3.4)	0.085
6 months	6.0 (4.0 to 8.0)	4.7 (3.2 to 6.2)	1.3 (-1.2 to 3.8)	0.312

Mean differences between groups were obtained with analyses of covariance with the baseline value as a covariate. Means at three and six months are estimated marginal means. HADS = Hospital Anxiety and Depression Scale.

Both groups evidenced a decrease of anxiety and depressive symptoms according to the HADS with a trend for a stronger effect in the paroxetine group. However, no statistically significant difference between paroxetine- and placebo-treated participants was detected at any time point (Table 3). There was no significant difference between the groups regarding body weight or BMI. Neither was any difference detected in serum glucose, serum c-peptide or serum SHBG.

A post hoc subgroup analysis of covariance was performed to elucidate whether the response to paroxetine was stronger among patients who fulfilled DSM-IV criteria for mild depression. In the subgroup of subjects with five to six DSM-IV depressive symptoms [n(placebo) = 4, n(paroxetine) = 5] a significant benefit of paroxetine was observable in serum glucose levels after six months (mean difference between groups = 3.88 mmol/l, 95%CI = 0.47 to 7.29, p = 0.032).

Fifteen of the 43 participants who initiated medication reported adverse events (Table 4). The adverse events were classified as mild with the exception of the combination of nausea and hypoglycaemia in one paroxetine-treated patient, which was classified as a moderate adverse effect.

**Table 4:** Number of patients in each group who reported adverse events.

	Placebo	Paroxetine
nausea	0	4
loss of appetite	1	4
headache	0	1
confusion	0	1
sedation	0	1
flatulence	1	0
hypoglycaemia	0	1
hyperglycaemia	1	0
erectile dysfunction	2	0
sweating	1	1

## Discussion

In our study we have shown that paroxetine had a beneficial effect on GHbA_1c _and quality of life when compared to placebo after three months of treatment. However, when treatment was continued the difference between groups was no longer significant.

Our primary outcomes, which we considered to be clinically significant, were a 0.8%-units difference in GHbA_1c _and a 10 points difference in SF-36 when comparing paroxetine to placebo. A statistically, significant improvement in SF-36 was reached after three months. However, by the end of the trial, the effect on quality of life was somewhat diminished and the difference between groups was no longer significant.

Our finding of a 0.59%-units difference in GHbA_1c _is not clinically significant according to our primary statement. Moreover even this minor effect was transient. We have no explanation as to why the effect is not sustained but a similar transient effect of fluoxetine on GHbA_1c _was seen in a study by Gray et al [18]. Our post hoc subgroup analysis suggests that a possible long term effect could be confined to those who fulfil criteria for clinical depression.

To our knowledge this is the first long-term double-blind placebo-controlled study where the effects of paroxetine on quality of life, metabolic control and mental health have been simultaneously assessed in type 2 diabetic subjects with mild depressive symptoms. According to our findings patients with sub-threshold depression do not benefit from a long-term treatment with paroxetine. This is relevant information for general practitioners who mostly treat the mildly depressed. It is possible that the generally used criteria for prescribing SSRIs are to loose and that GPs should use other methods for treating patients with sub-threshold depression.

The study was built upon our previous smaller and shorter single-blind pilot study [12], in which we found a significant improvement in an indicator of insulin sensitivity, SHBG, in the treatment group when compared to the control group, and a trend towards better glycemic control in the paroxetine group than in the placebo group. The previous study had several weaknesses, i.e. small sample size, (N = 15), lack of double-blindness, short duration (10 weeks), and a rather good baseline metabolic control (mean GhbA_1c _7.5 %). We aimed at overcoming the weaknesses of the pilot trial by performing the current 6-month double-blind trial with stricter inclusion criteria for glyacemic control (> 7 % vs. = 6.5 % in the previous trial). We did not replicate our previous finding of a differential change in SHBG values in the paroxetine group when compared to placebo.

## Conclusion

Our results do not exclude the possibility that long-term paroxetine treatment may have an either beneficial or harmful effect on quality of life or mental health status in diabetic out-patients with minor depressive symptoms, but the harmful effect observed in our study was less than modest and our results indicate that the possible beneficial effect is modest and transient. Routine paroxetine treatment of diabetics with sub-threshold depression is not warranted.

## Competing interests

GlaxoSmithKline, the manufacturer of paroxetine, donated paroxetine and identical placebo, and provided a grant to the researchers.

## Authors' contributions

JE and KW designed the study. MP carried out the interviews and performed outcome assessment. KW coordinated writing of the report. JE was the principal investigator. All authors read and approved the final manuscript.

## Pre-publication history

The pre-publication history for this paper can be accessed here:


